# Genome Editing in Agriculture: Technical and Practical Considerations

**DOI:** 10.3390/ijms20122888

**Published:** 2019-06-13

**Authors:** Julia Jansing, Andreas Schiermeyer, Stefan Schillberg, Rainer Fischer, Luisa Bortesi

**Affiliations:** 1 Aachen-Maastricht Institute for Biobased Materials (AMIBM), Maastricht University, Brightlands Chemelot Campus, Urmonderbaan 22, 6167 RD Geleen, The Netherlands; julia.jansing@maastrichtuniversity.nl; 2 Fraunhofer Institute for Molecular Biology and Applied Ecology IME, Forckenbeckstrasse 6, 52074 Aachen, Germany; andreas.schiermeyer@ime.fraunhofer.de (A.S.); stefan.schillberg@ime.fraunhofer.de (S.S.); 3 Indiana Biosciences Research Institute (IBRI), 1345 W. 16th St. Suite 300, Indianapolis, IN 46202, USA; rfischer@indianabiosciences.org

**Keywords:** base editors, oligonucleotide-directed mutagenesis, precision breeding, programmable nucleases, sequence-specific nucleases

## Abstract

The advent of precise genome-editing tools has revolutionized the way we create new plant varieties. Three groups of tools are now available, classified according to their mechanism of action: Programmable sequence-specific nucleases, base-editing enzymes, and oligonucleotides. The corresponding techniques not only lead to different outcomes, but also have implications for the public acceptance and regulatory approval of genome-edited plants. Despite the high efficiency and precision of the tools, there are still major bottlenecks in the generation of new and improved varieties, including the efficient delivery of the genome-editing reagents, the selection of desired events, and the regeneration of intact plants. In this review, we evaluate current delivery and regeneration methods, discuss their suitability for important crop species, and consider the practical aspects of applying the different genome-editing techniques in agriculture.

## 1. Introduction

The precise modification of pre-selected sequences in the plant genome is the holy grail of plant breeding because it allows the rapid introduction of genetic diversity and accelerates the generation of improved varieties, especially in polyploid crops, which otherwise need to undergo lengthy programs of crossing and screening. The development and application of genome-editing tools in recent years has, therefore, revolutionized basic research in plant biology and the generation of new plant varieties. Although the molecular basis of genome editing in plants has been comprehensively discussed in the literature, the practical aspects have received comparatively little attention. Accordingly, in this article, we emphasize some of the technical and practical aspects of genome-editing technologies for crop improvement.

## 2. Genome-Editing Tools

### 2.1. Programmable Sequence-Specific Nucleases

There are three major genome-editing techniques categorized by mechanism of action, and by far the most commonly used in plants is the targeted generation of DNA double strand breaks (DSBs) using programmable sequence-specific nucleases followed by DNA repair via one of two major endogenous pathways [1,2]. The error-prone non-homologous end joining (NHEJ) pathway joins DNA ends and is often accompanied by the insertion or deletion (indel) of short stretches of nucleotides at the junction. When the DSB is within a coding sequence, the resulting mutation often causes a loss of function, which can be exploited to determine gene functions (reverse genetics) or to abolish undesirable traits to improve crops. In contrast, homology-directed repair (HDR) is a more accurate pathway but it requires the presence of a DNA template with homology to the sequences upstream and downstream of the DSB [3]. By providing a donor DNA template with homology to the target site, this pathway can be exploited for the precision engineering of endogenous genes or for the addition of genes or other sequences at predetermined genomic loci.

The targeted induction of DSBs is achieved using programmable nucleases. The most common nucleases for genome editing are zinc finger nucleases (ZFNs), transcription activator-like effector nucleases (TALENs), and Cas9, the latter being part of the clustered regularly interspaced short palindromic repeat (CRISPR)/Cas9 system. The three classes of nucleases differ in structure, activity, and enzymatic mechanism, resulting in differences in target selection, efficiency, specificity, and mutation signature. We will focus on these practical aspects of each nuclease class and refer readers to other comprehensive reviews for information on the history of each system and the development of improved variants.

ZFNs and TALENs are artificial enzymes consisting of a series of DNA-binding domains (zinc fingers and TAL effector domains, respectively) fused to the sequence-independent catalytic domain of the type IIS restriction endonuclease FokI. In its natural form, FokI must dimerize to cleave DNA, so both ZFNs and TALENs also function as dimers and, like FokI, generate DSBs with 5′ cohesive overhangs.

One zinc finger module binds a triplet of nucleotides. A single zinc finger would, therefore, lack the specificity to bind a unique genomic target, but typical constructs contain three or four fingers, which translates to a ZFN target sequence of 18–24 bp (9–12 bp per half-site). This length is sufficient to target unique sites even in the large genomes of higher eukaryotes. The target site can be any length in principle, but the context-dependent assembly of ZFN modules limits the size in practice [4]. Unlike zinc fingers, each TAL effector recognizes a single nucleotide. Engineered TAL effector domains are typically designed to recognize 15–30 nucleotides, making a total of 30–60 nucleotides for one TALEN pair. Therefore, TALENs are generally considered to bind with greater specificity than ZFNs, even though larger TAL effector domains are more likely to tolerate mismatches [5]. To further minimize off-target events and associated cellular toxicity, the FokI nuclease dimerization interface has been engineered to force heterodimer formation, and this variant is routinely used in ZFNs and TALENs [6,7,8].

In terms of target choice, publicly available ZFN module libraries can be used to prepare functional ZFN pairs that match one site in every ~100 bp of random genomic DNA [9]. This means that target sites are available for the disruption of most conventional genes, but it may be more difficult to target small non-coding RNA genes, short regulatory regions, or genomic sites that happen to fall between the available targets. In contrast, the selection of target sites for TALENs is restricted only by the requirement for a thymidine at the first position, so TALEN targets can be found on average every 35 bp [10].

The half-sites recognized by ZFNs are typically separated by 6–8 bp depending on the design of the fusion protein [11]. For TALENs, the spacer length is usually 15–30 bp. In the absence of a repair template, both nucleases predominantly generate indels a few base pairs in length [12], but ZFNs tend to produce a larger proportion of insertions than deletions, probably because the short overhangs resulting from the shorter spacers can be more efficiently filled in before ligation [13]. The generation of more insertions than deletions is a potential drawback in terms of regulation because the added DNA might be considered a novel sequence, but this aspect has yet to be explored in practice [14].

The number of publications reporting the use of ZFNs and TALENs in plants is rather limited and tends to favor TALENs [15], but the editing efficiency that can be achieved with both types of nuclease (i.e., the number of correctly edited events as a proportion of all attempts) appears to be similar. Under appropriate experimental conditions, high editing efficiencies are possible with both ZFNs and TALENs. In maize (*Zea mays*), for example, the reported efficiency of gene targeting with ZFNs was 40% [16] compared to 40–60% with TALENs [17]. Lower efficiencies reported in other cases can probably be attributed to the experimental conditions and/or the choice of target sequence. TALENs are significantly larger and more repetitive than ZFNs, which can affect the efficiency of intracellular nuclease delivery and in turn the overall efficiency of editing. The requirement for larger constructs can also exclude the use of viral vectors, which have a limited insert capacity.

Where specifically investigated, ZFNs did not induce off-target mutations in plants [16] and TALENs only rarely caused cytotoxicity due to off-target cleavage [18,19]. However, the authors in each case only looked for mutations at predicted off-target sites, whereas genome-wide screening is required for unbiased analysis [20]. The identification of potential off-target sites for ZFNs and TALENs is challenging (particularly for ZFNs) because sequence specificity is conferred by protein–DNA interactions that are often context-dependent and difficult to predict. A final drawback of ZFNs and TALENs is the requirement of different dimeric proteins specific for each target site, which limits their practical use for multiplexing (the simultaneous introduction of DSB at multiple sites) unless the target sites are closely related [21].

The most recent addition to the toolbox of programmable nucleases (and the most widely used in plants) is Cas9 from *Streptococcus pyogenes* (SpCas9), which is part of the CRISPR/Cas9 system [22,23]. Cas9 is a monomeric nuclease that forms a ribonucleoprotein (RNP) complex with a chimeric guide RNA (gRNA). The latter confers sequence specificity by providing a 20-nucleotide sequence complementary to the target site (known as the protospacer). The Cas9 enzyme possesses two nuclease domains, each cleaving one strand of the target sequence three nucleotides upstream of the protospacer adjacent motif (PAM) to generate blunt ends [24]. The only constraint for the design of the gRNA is the PAM, which needs to be present at the 3′ end of the target sequence. For SpCas9, this sequence is defined as 5′-NGG-3′ [25]. The in silico analysis of plant nuclear genome sequences (including monocots and dicots) has identified 5–12 NGG-PAMs on average for every 100 bp [26]. Given the relatively short target sequence and the high frequency of PAM sites, it can be challenging to identify specific targets, especially in crops with large and highly repetitive genomes such as maize. Cas9 variants from other species such as *S. aureus* (SaCas9, recognizing the less frequent NNGRRT-PAM [27]) and SpCas9 mutants that recognize non-canonical PAMs, can broaden the range of CRISPR/Cas9 genome-editing targets in plants with complex genomes [28].

One major advantage of CRISPR/Cas9 over ZFNs and TALENs is that the Cas9 enzyme does not need to be engineered at the protein level to recognize different targets. Target specificity is conferred entirely by the spacer region of the gRNA, and the sequence can be modified using standard molecular biology methods [29]. Given the simplicity of the CRISPR/Cas9 system, it has superseded ZFNs and TALENs in research applications and a large body of literature has accumulated, describing the use of this system in many different plant species. Furthermore, by providing multiple gRNAs simultaneously, the CRISPR/Cas9 system can be used to target different genes in parallel, including unrelated genes [30,31,32,33].

The CRISPR/Cas9 system is at least as efficient as ZFNs and TALENs in cereal crops [12] and for most species, there are examples of editing efficiency approaching 100% [34]. In general, targeting one gene at two positions increases the overall mutation frequency and allows the recovery of homozygous mutants in one generation [33,35]. The ease of multiplexing with the CRISPR/Cas9 system is therefore an advantage for the generation of knockouts using this dual-gRNA approach. In contrast to ZFNs and TALENs, Cas9 generates blunt DSBs that are typically repaired by the formation of small (usually 1-bp) indels, leading to the frequent recovery of frameshift mutants when the target site is within an exon [34].

More recently, the CRISPR/Cas12a (originally named Cpf1) system was discovered and also developed into a genome-editing tool [36,37]. Cas12a recognizes a TTTN-PAM preceding the target sequence, which facilitates the targeting of AT-rich regions and reduces the likelihood of off-target mutations in GC-rich genomes, adding more flexibility to the application of CRISPR/Cas systems in plants. Cas12a produces a staggered cut with 5′ overhangs of five nucleotides more distal to the PAM, which in turn favors the generation of deletions 6–13 bp in length, considerably larger than the mutations generated by Cas9 [38]. These features further broaden the sequence space that can be targeted using CRISPR/Cas systems. A recent study in rice suggests that several Cas12a variants are temperature sensitive and that editing efficiencies can be increased substantially when plants are grown at 28 °C instead of 22 °C [39].

The frequency of off-target mutations generated by CRISPR/Cas systems has been raised as a concern, even though the specificity of CRISPR/Cas9 in plants appears to be higher than in mammals. Unexpected DSBs have been reported for only a minority of gRNAs, even when whole-genome sequencing has been used to screen for off-target mutations [40]. Several high-fidelity variants of SpCas9 and SaCas9 have also been developed, along with strategies to increase the length of the recognition sequence by engineering an inactive Cas9 enzyme fused to the FokI nuclease domain, similar in principle to ZFNs and TALENs (reviewed in [41]). In most cases, the likelihood of off-target mutations can be reduced by optimizing the experimental setup (discussed in more detail below) and by the careful design of the gRNA. Recently, a further major improvement in the prediction of off-target sites in plants has been achieved by developing better alignment algorithms and taking into account rare but important off-target sequences [42].

### 2.2. Base Editors

Given the unpredictable outcome of DSB repair via NHEJ and the low efficiency of HDR, researchers have sought new methods to introduce point mutations without the need for DSBs or donor templates, resulting in the development of base editors as a new tool for genome editing [43]. To achieve the conversion of cytidine to uridine, a cytidine deaminase was fused via a linker to the N-terminus of a semi-active version of Cas9, in which one of the nuclease domains is inactivated by the mutation D10A to generate a nickase (nCas9). The combination of the nickase and cytidine deaminase triggers DNA mismatch repair, resulting in the targeted conversion of a C:G base pair to T:A. In addition to base substitutions, indels can sometimes occur as a consequence of the DNA nicks induced by nCas9 on the non-edited strand [44]. The first generation of base editors had an editing window of a few base pairs, causing unwanted bystander mutations [45]. By controlling the length and flexibility of the linker, a high-precision base editor has recently been reported that can selectively edit a single C at a specific position with high accuracy and efficiency [46]. Recently, base editors based on Cas9 variants with high fidelity and relaxed PAM requirements have been developed [47,48].

The conversion of A:T to G:C is more challenging because there are no known adenine deaminases that act on DNA, but this has been addressed by the directed evolution and engineering of tRNA adenosine deaminases to accommodate DNA substrates [49]. A tRNA adenosine deaminase has recently been adapted for base editing in plants by directed evolution, achieving A:T to G:C conversion at frequencies of up to 7.5% in protoplasts and 59.1% in regenerated rice (*Oryza sativa*) and wheat (*Triticum aestivum*) plants [50]. This base editor has a deamination window extending from positions 4 to 8 of the protospacer. In mouse embryos, unbiased mutation screening has recently shown that base editing causes off-target mutations at a 20-fold higher frequency than Cas9, raising concerns over the use of this technology for sensitive applications [51]. No significant conversion has been observed at predicted off-target sites in plant DNA [52,53,54,55], but genome-wide screening using unbiased methods is needed to properly assess the specificity of base editors. A recent study reported low but detectable off-target tRNA adenosine deaminase activity against cellular RNAs in mammalian cells and described base editor enzymes with reduced DNA and RNA off-target activity [56]. Even so, base editing is more efficient for the introduction of point mutations than alternative approaches such as TILLING or gene targeting by homologous recombination [55]. Multiplexed base editing without any loss of efficiency has been reported in mammals [57,58] but not yet in plants.

### 2.3. Oligonucleotide-Directed Mutagenesis

Mutagenic DNA oligonucleotides 20–200 nucleotides in length have been delivered into plant cells to introduce point mutations in target genes, an approach known as oligonucleotide-directed mutagenesis (ODM). This technique harnesses the endogenous HDR pathway to correct mismatches generated by pairing the exogenous oligonucleotide, which carries the desired sequence, to its near-complementary target site in the genome. The oligonucleotide therefore acts as both a mutagen and a DNA repair template. Mutagenesis can be achieved using standard single-stranded DNA oligonucleotides (ssODNs) but these have a short intracellular half-life, and their efficiency has therefore been improved by stabilizing modifications. The modified variants include chimeraplasts (duplexes of DNA and methylation-modified RNA), phosphorothioate-modified ssODNs, and ssODNs with a 5′ Cy3 label and a 3’idC reverse base modification [59].

The efficiency of ODM is generally rather low and positively correlates with oligonucleotide length, at least in the case of ssODNs, where increasing the length to 200 nucleotides achieved precise editing frequencies of up to 0.05% at a transgenic locus in Arabidopsis (*Arabidopsis thaliana*) protoplasts [59]. Chimeraplasts did not increase mutation frequencies above the level of spontaneous mutations in tobacco (*Nicotiana tabacum*) or rapeseed (*Brassica napus*) [60]. However, targeted mutation frequencies could be enhanced by ODM in concert with nonspecific DSB-inducing reagents such as antibiotics, or sequence-specific nucleases such as TALENs and CRISPR/Cas9 in Arabidopsis and flax (*Linum usitatissimum*) [61]. Off-target mutations could be generated by ODM due to oligonucleotide recombination or off-target mismatch repair, but such events have not been reported. ODM theoretically allows multiplexing, i.e., multiple conversions at several targets within a single gene or the simultaneous conversion of multiple targets in a single cell.

## 3. Outcomes of Genome Editing

**Figure 1:**
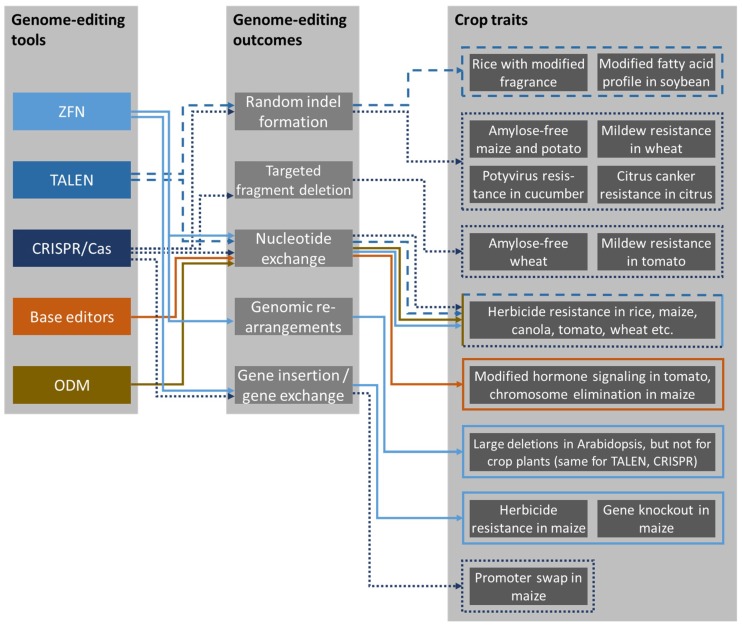
Overview of genome-editing tools, the possible genetic outcomes in each case, and examples of crop traits generated using these tools. The colored arrows and boxes link published crop trait examples with the associated genome-editing tool and outcome.

There are five classes of genome modifications that can be induced using the tools described above, although not all outcomes are possible with all tools. All five types of modification have been reported in crop plants, and each is discussed below with some examples of gene modifications associated with relevant traits and outcomes. Figure 1 presents an overview of the different genome-editing tools, the potential outcomes of genome editing in each case, and examples of modified crop traits.

### 3.1. Random Indel Formation

When a single DSB is induced in higher plants, the most frequent outcome is the formation of indels around the cleavage site. When the indels introduced by erroneous break repair lead to a frameshift—which theoretically is the case for 2/3 mutations within the open reading frame of a gene—the resulting transcript usually contains premature stop codons. These are recognized by endogenous quality control processes and can initiate nonsense-mediated mRNA decay and thus result in the functional knockout of the mutated gene [62]. This is the simplest form of genome editing. Most applications of sequence-specific nucleases (particularly the CRISPR/Cas9 system) have concerned the generation of indels to achieve gene knockout. Some examples are discussed below.

TALENs have been used to knock out the rice *OsBADH2* gene encoding a betaine aldehyde dehydrogenase. Homozygous plants of the non-fragrant rice variety Nipponbare with mutations in this gene emitted a desirable fragrance due to the accumulation of 2-acetyl-1-pyrroline, similar to natural fragrant varieties such as Indian Basmati and Thai Jasmine, which usually trade at higher market prices [63]. TALENs have also been used in soybean (*Glycine max*), a staple crop cultivated for the high protein and oil content of its seeds, to modify the fatty acid profile. Soybean oil naturally contains a high percentage of the polyunsaturated fatty acids linoleic acid (18:2) and linolenic acid (18:3) making it sensitive to oxidation. Therefore, soybean oil has been partially hydrogenated to reduce the quantity of polyunsaturated fatty acids, but this process generates trans-fatty acids with negative health effects. Such trans-fatty acids resulting from the partial hydrogenation of food ingredients have therefore been banned by the FDA [64,65]. To reduce the levels of linoleic and linolenic acid, the genes encoding fatty acid desaturases FAD2 and FAD3 were disrupted using TALENs [66,67]. Homozygous soybean plants carrying the knockout alleles *fad2-1a*, *fad2-1b,* and *fad3a* produced oil with <3% linoleic acid and linolenic acid (compared to 51% and 8%, respectively, in wild-type plants) but much higher (> 80%) levels of oleic acid (18:1) than wild-type plants (23%). Due to the lower levels of polyunsaturated fatty acids, the seed oil from these events is less sensitive to oxidation and does not require processing by partial hydrogenation.

Starch is the main carbohydrate storage molecule in plants and has many applications in addition to its use in food and beverages. Naturally, starch occurs in two different forms (amylose and amylopectin), but for certain industrial applications, it is desirable to have a homogenous starch composition rather than a mixture. Therefore, efforts have been made to engineer maize and potato (*Solanum tuberosum*) plants that are devoid of amylose and accumulate only amylopectin, a phenocopy of the natural maize mutant *waxy* [68]. Granule-bound starch synthase I (GBSSI) is the key enzyme required for amylose synthesis, and has been targeted in tetraploid potato plants by transfecting protoplasts with preassembled Cas9/gRNA RNPs [69]. Using this approach, 2.3% of all regenerated shoots contained mutations in all four alleles of the *GBSSI* gene, highlighting the power of sequence-specific nucleases in polyploid crops.

Engineering pathogen resistance is another major goal of crop improvement. Sequence-specific nucleases have been used successfully in this context, including the generation of wheat plants resistant to *Blumeria graminis* f. sp. *tritici*, the fungal pathogen responsible for powdery mildew disease. Wheat plants with a non-functional mildew resistance locus (*MLO*) are naturally resistant to the pathogen, but the locus has been difficult to target using traditional mutagenesis techniques because bread wheat is an allohexaploid species with six *MLO* homeoalleles at three loci (*TaMLO-A1*, *TaMlo-B1,* and *TaMlo-D1*). Given the ability of single TALEN pairs and single gRNAs to target multiple conserved sequences simultaneously, both methods have been used to generate knockouts of all six homeoalleles (*tamlo-aabbdd*) resulting in mildew-resistant wheat varieties [70]. Similarly, CRISPR/Cas9 technology has been used in cucumber (*Cucumis sativus*) to knock out the gene encoding translational initiation factor eIF4E, which is essential for the Potyvirus infection cycle, generating plants with broad virus resistance [71]. Finally, disease resistance has also been achieved by using CRISPR/Cas9 in Wanjincheng orange (*Citrus sinensis* Osbeck) plants to target the promoter of the susceptibility gene *CsLOB1*, resulting in plants with enhanced resistance to citrus canker [72].

### 3.2. Targeted Fragment Deletion

When two or more DSBs are induced within the same gene, it is possible to achieve gene knockout via the targeted deletion of the intervening sequence. Although theoretically possible with any sequence-specific nuclease, this form of genome editing is most easily achieved with the CRISPR/Cas9 system, which is highly amenable to multiplexing. There are two main advantages associated with this approach: First, when deleting a larger fragment of DNA, the chances of disrupting gene function are higher; and second, analysis of the events can be greatly simplified when the difference in size between wild-type and mutated PCR amplicons can be assessed simply by gel electrophoresis or melt analysis, rather than fragment digestion or sequencing. The drawback is that the frequency of fragment deletion depends on the efficiency of the nucleases, and often results in indels at one or both cleavage sites rather than removal of the intervening sequence. Increasing the number of gRNAs can also increase the number of potential off-target sites.

In practical examples of this approach, scientists at Pioneer Hi-Bred used CRISPR/Cas9 to inactivate the enzyme GBSSI in elite maize germplasm by deleting a large portion of the *wx1* locus using two gRNAs [73]. Disease resistance has also been achieved using this approach, for example in the tomato (*Solanum lycopersicum*) *Mlo1* locus, which was targeted using two gRNAs to generate a 48-bp deletion. Homozygous and biallelic deletion mutants were resistant to the powdery mildew fungus *Oidium neolycopersici* [74].

### 3.3. Targeted Nucleotide Exchange

Precise nucleotide exchanges (often described as gene conversion or allele replacement events if they are driven by homologous recombination) can be used not only to achieve gene knockout (e.g., by introducing an early termination codon), but also to generate gain-of-function mutations or regulatory mutations that modulate gene expression.

All three of the genome-editing tools described above can be used to achieve nucleotide exchanges, but unassisted sequence-specific nucleases generate such mutations only rarely, and the replacement is random. Therefore, targeted nucleotide exchange using sequence-specific nucleases is generally achieved by providing a repair template of either single-stranded or double stranded DNA carrying the desired allele, to promote DSB repair via the endogenous HDR pathway (i.e., gene conversion). Higher plants overwhelmingly favor NHEJ over HDR, so the frequency of desired point mutations is typically rather low. The few examples that have been reported mainly concern the generation of a selectable trait such as herbicide resistance to favor the isolation of precise editing events. TALENs and the CRISPR/Cas9 system have been used for nucleotide exchange in the rice gene encoding acetolactate synthase (*ALS*), generating plants resistant to bispyribac-sodium [75]. Furthermore, ZFNs have been used for nucleotide exchange in the wheat *ALS* gene to confer resistance to imidazolinone herbicides [76]. By delivering the CRISPR/Cas9 components either as DNA or RNP in combination with single-strand oligonucleotides, the maize *ALS2* gene was also edited and chlorsulfuron-resistant plants were obtained [77,78].

ODM can also be used to promote nucleotide exchange and this is the basis of the proprietary Rapid Trait Development System developed by Cibus, Inc. (www.cibus.com). Using this technology, Cibus has already produced a commercial sulfonylurea-tolerant canola line (SU Canola) and several other similarly modified crops are in the pipeline: Rice, flax, potato, wheat, maize, cassava (*Manihot esculenta*), and peanut (*Arachis hypogaea*). The ODM approach can also be combined with sequence-specific nucleases so that the ssODN acts as the repair template for the DSB. This strategy has been used to introduce nucleotide exchange in the flax *EPSPS* gene encoding 5′-enolpyruvylshikimate-3-phosphate synthase, conferring glyphosate resistance [61]. Notably, the editing efficiency was sufficient to allow the regeneration of edited plants without selection.

Another way to achieve targeted nucleotide exchange is the use of base editors. For example, base editors incorporating cytidine deaminase have been used to generate rice varieties with precise exchanges in genes conferring herbicide resistance [53] and senescence-related traits [55], and tomato plants with the anticipated modification in a gene controlling hormone signaling [53]. In maize, the same approach has been used to introduce single-nucleotide exchanges in the CENP-A targeting domain of CENH3, a protein required for uniparental chromosome elimination during the production of double haploids [55]. More recently, base editors incorporating adenosine deaminase have been used to modify the pathogen-responsive phosphorylation site in several endogenous rice genes [54], to generate rapeseed plants with a delayed flowering phenotype [52], and to confer herbicide resistance in rice [50].

### 3.4. Genomic Rearrangements

The introduction of two DSBs in the same gene can lead to targeted DNA fragment deletion as discussed above. However, if the two DSBs are further apart on the same chromosome, the outcome can be a major cytogenetic deletion or inversion, and if the DSBs are on different chromosomes, then the resolution can generate a translocation event. Although rare, such genomic rearrangements offer interesting opportunities to breeders: Large deletions could be used to remove entire gene clusters, translocations can create new linkages between interesting traits or break undesirable linkages, and inversions on homologous chromosomes could help to transfer traits from wild relatives to elite cultivars [79].

Large chromosomal deletions (up to ~9 Mb) and inversions (~10.6 kb) have been generated in Arabidopsis using ZFNs [80]. In rice, large chromosomal deletions of up to 245 kb were generated in protoplasts and regenerated plants using CRISPR/Cas9, but their transmission to the T1 generation was not investigated [81]. Furthermore, a recent preprint reports the CRISPR/Cas9-mediated duplication and meiotic transmission of a 2.3-kb DNA fragment in Arabidopsis [82]. In rice, the inversion of a 1.3-kb DNA fragment was achieved with a frequency of 1% using TALENs [83], and the inversion of a ~300-bp fragment between two DSB generated by CRISPR/Cas9 was observed in one of nine tested gRNA pairs [84]. Although theoretically possible, heritable larger inversions and translocations have not yet been reported in crop species.

### 3.5. Gene Insertion and Gene Exchange

The DSBs generated by sequence-specific nucleases can also be exploited to insert larger sequences, including complete genes, either by direct fusion to the free ends (NHEJ) or by recombination with a construct in which the input gene is flanked by homology regions matching the target site. The precise insertion of transgenes in this manner eliminates the risks associated with random insertion (position effects, interference with/mutation of endogenous genes) and can be used to generate either cisgenic or transgenic plants.

Sequence-specific nucleases can also simplify breeding strategies by facilitating trait stacking to optimize crop performance (e.g., herbicide tolerance and pest resistance in the same variety). As the number of genes to be incorporated in the germplasm increases, the associated breeding programs become more complex. It is therefore desirable to integrate multiple transgenes at defined loci to facilitate subsequent breeding efforts. This has been addressed by equipping transgene cassettes with unique sequences that act as landing pads for ZFNs in subsequent rounds of editing [85]. If the first transgene carries a unique sequence corresponding to a ZFN target site, then the transgenic event can be super-transformed with another transgene and a suitable ZFN construct such that the incoming transgene is added in tandem to the incumbent transgene. If the second transgene also carries a unique landing pad (different to the first), the process can be repeated any number of times. Using this approach, the herbicide resistance gene *aad1* coding for aryloxyalkanoate dioxygenase [86] has been integrated adjacent to an existing phosphinothricin acetyltransferase (*pat*) transgene in maize [85].

In an ingenious approach, the targeted integration of 7.1-kb and 16.2-kb multigene constructs was demonstrated in soybean embryos. The constructs were delivered as circular donor DNAs together with ZFN constructs targeting both the endogenous fatty acid desaturase gene *FAD2-1a* and the donor DNA. Following cleavage by the ZFNs, microhomology between the compatible overhangs in the target and the donor promoted the seamless insertion of the multigene constructs into the *FAD2-1a* gene by NHEJ [87]. By simultaneous cleavage at the 5′ and 3′ ends of a target gene and the delivery of an appropriately designed donor template, gene exchange can also be achieved by HDR [88]. For example, ZFNs have been used to knock out the gene encoding inositol-1,3,4,5,6-pentakisphosphate 2-kinase (IPK) in maize following this strategy [16]. The IPK enzyme catalyzes the final step in the pathway leading to the synthesis of phytate, the main phosphate storage molecule in seeds and grains. To achieve gene inactivation, IPK-specific ZFNs and a donor DNA encoding the marker gene *pat* flanked by homology arms were co-delivered, and the *IPK1* gene was disrupted by the integration of *pat*, enabling the identification of targeted events by screening seedlings for bialaphos resistance. Using a similar approach, CRISPR/Cas9 has been used to replace the weak endogenous maize *ARGOS8* promoter with a more active promoter sequence, producing varieties with increased grain yield under drought stress conditions in the field [89].

## 4. Delivery Methods

When deciding which of the three major genome-editing tools to use, one major issue is how and in what form the tool will be delivered to the plant cell. For most plant species, more than one delivery method can be used, and the genome-editing tools can be supplied as DNA, RNA, proteins, or RNPs. There are certain technique-specific limitations regarding the choice of material: ODM requires the use of oligonucleotides, and only CRISPR/Cas9 and its derivatives feature an RNA component allowing RNP delivery. In this section, we discuss the delivery methods currently available for genome-editing tools, dividing them into those suitable for DNA, RNA, proteins, and RNPs. When first mentioned, the delivery methods are briefly explained and practical considerations for their use in genome editing are discussed.

### 4.1. DNA

Most, if not all, methods for the delivery of genome-editing reagents have evolved from methods originally developed for transgenesis, which involved the delivery of recombinant DNA. Here researchers have their pick and can select the best-established method in the plant species of interest or the method best suited for the experiment at hand. The methods discussed in this section are suitable for the delivery of constructs encoding programmable sequence-specific nucleases and base editors. Oligonucleotides for ODM also comprise or contain DNA, but for the purpose of this review, they are discussed in the RNA section because the requirements for their delivery are more closely related to those for RNA reagents.

#### 4.1.1. Agrobacterium-Mediated Transformation

The soil-dwelling plant pathogen *Agrobacterium tumefaciens* has the natural ability to transfer a segment of DNA (T-DNA) from a resident plasmid into plant cells and subsequently facilitate the integration of that DNA into the nuclear genome, resulting in the development of plant tumors (crown galls) that shelter the bacteria and produce metabolites they can use as an energy source. By replacing the bacterial genes normally found on the T-DNA, this method can be used to introduce transgenes into any plants in the Agrobacterium host range [90]. To streamline this process in the laboratory, several disarmed binary vector systems have been developed [91]. Disarmed in this context means that the genes that induce tumor growth and metabolic activity are removed [92], and binary means that the T-DNA is located on a small shuttle vector to facilitate cloning in *Escherichia coli* and maintenance in Agrobacterium, whereas the virulence genes needed for DNA transfer are located on a second helper plasmid [90].

T-DNA transfer requires the bacterial and plant cells to be brought into close contact, which can be achieved by syringe or vacuum infiltration of leaves [93], the co-cultivation of Agrobacterium and suitable plant tissues [94], cells [95], or protoplasts [96], by floral dip [97] or by spraying [98]. However, the efficiency of these methods is species-dependent: The infiltration of leaves does not work well in monocots [99], floral dip is used almost exclusively with Arabidopsis [100], and the most competent tissue for transformation also differs by species and, in some cases, by cultivar. Regardless of the transfer method, Agrobacterium can routinely introduce large T-DNAs of ~25 kb [101,102], which is usually sufficient to carry the *S. pyogenes cas9* gene (~4 kb), several gRNA genes, and the primary transgene of interest. However, binary plasmids larger than 10–15 kb are difficult to maintain in standard *E. coli* strains [103]. One advantage of Agrobacterium-mediated transformation is that the quality of integration events is often high: The T-DNA is usually intact and there is a high frequency of single-copy/low-copy-number integration events [104]. The main drawback is the host specificity of available Agrobacterium strains: Dicots are generally much more amenable than monocots, although the transformation of recalcitrant monocots is possible using specialized techniques to increase competence [105].

In the context of genome editing, Agrobacterium is used most often to generate transgenic plants that have one or more T-DNA copies stably integrated into the genome, allowing the constitutive or inducible expression of a primary transgene along with a selectable marker [106]. Efficient transformation and regeneration protocols exist for many plant species, making this approach particularly suitable for the delivery of DNA constructs encoding the genome-editing reagents and the subsequent regeneration of transgenic plants. Interestingly, it is also possible to use Agrobacterium to transiently express the CRISPR/Cas9 components in tobacco leaves, which allows the recovery of non-transgenic genome-edited plants: 17% of the genome-edited plants recovered using this method were non-transgenic [107]. However, both the stable transformation and transient expression approaches come with a trade-off between on-target and off-target mutations. Stable transformation leads to the sustained expression of the genome-editing components (e.g., Cas9 and gRNA in the CRISPR/Cas9 system), which increases the efficiency of on-target mutations [108] but also risks the accumulation of mutations at off-target sites [109]. In contrast, transient expression achieves a lower frequency of both on-target and off-target mutations because the components are available for a shorter time, but the payoff is that no foreign DNA is integrated into the genome [110].

Genome-editing tools can also be delivered using deconstructed viral vectors, including those based on single-stranded DNA viruses of the Geminivirus family [111,112], or single-stranded RNA viruses such as Tobacco rattle virus [113], Tomato mosaic virus [114], and Tobacco mosaic virus [115]. Such deconstructed virus genomes generally lack the ability to move from cell to cell and are therefore incorporated into the T-DNA and introduced into the host plant by agroinfiltration [113]. The ability of viral vectors to replicate is particularly interesting in the context of genome editing because high copy numbers of genes or HDR templates can be generated in vivo to achieve higher editing efficiencies in combination with a targeted DSB [111]. The advantage of RNA viruses is that the replicated RNA genome does not integrate, but it also means that they cannot be used to amplify DNA repair templates. Their limited cargo capacity is also a drawback, preventing the delivery of large genes such as *SpCas9* [111,116]. Theoretically, viral vectors could be delivered to plant cells or tissues without Agrobacterium, but for genome-editing purposes, viral vectors are routinely delivered as part of a T-DNA construct [111,117,118,119].

#### 4.1.2. Particle Bombardment

Particle bombardment is a physical transformation method that is particularly suitable for species beyond the Agrobacterium host range. Tungsten or gold microparticles are coated with DNA, RNA, protein, or RNPs, and accelerated by gas pressure into cells [120]. In the aqueous environment of the cell, the nucleic acid cargo can elute from the microparticles and integrate into the genome, or remain as an extrachromosomal construct (transient expression). The advantage of particle bombardment is its genotype independence [105] and versatility, which means that it can be used to transform a broad range of plant species in the form of different explants. However, the quality of the integration events is usually lower than Agrobacterium-mediated transformation and rarely results in clean single-copy integration of the DNA construct [104,105]. Both circular and linear DNA can be introduced into plant cells by particle bombardment, and the co-delivery of different DNA fragments or plasmids is quite efficient because they can be coated onto the same microparticles [121,122]. A recent study in wheat demonstrated the ZFN-mediated precise editing of the three homoeologous *ALS* genes following bombardment with the ZFN construct and a short dsDNA donor with compatible overhangs [76].

#### 4.1.3. Protoplast Transfection

Most techniques that are routinely used for the introduction of DNA into mammalian cells are unsuitable for intact plant cells because the cell wall is a physical barrier. However, if the cell wall is removed enzymatically, the resulting protoplasts can be transfected using techniques similar to those used with mammalian cells. The protoplasts can resynthesize their cell walls and proliferate in suspension, but the regeneration of intact plants is a severe bottleneck and can only be achieved routinely in a few model species [123,124,125]. Nevertheless, protoplasts are useful for testing the efficiency of different genome-editing reagents, at least in species that readily allow protoplast isolation [126]. Although there are many different transfection reagents for mammalian cells, the transfection of plant protoplasts is usually achieved using polyethylene glycol (PEG), which allows the efficient introduction of both circular and linear DNA by permeabilizing the plasma membrane [124,127]. Specialized vectors are not required because the transfer mechanism is physical rather than biological, and multiple plasmids can therefore be introduced simultaneously. The introduced DNA can be expressed transiently or it may integrate into the genome, resulting in high frequencies of stable transformation [88,123]. For example, Nicolia et al. [128] delivered a TALEN-expressing plasmid into potato protoplasts with a transformation efficiency of ~39% and used PCR to screen for transgene-free genome-edited callus. They targeted the *ALS* gene and found 11%–13% of callus clones and 10% of regenerated shoots were mutated at the target site [128].

#### 4.1.4. Electroporation

The electroporation of protoplasts involves the establishment of a strong electric field that changes the permeability of the cell membrane by opening transient pores, allowing the diffusion of nucleic acids [129]. Electroporation has been used for DNA delivery to the protoplasts of various crop species [129,130,131], and for gene targeting in tobacco BY-2 cells [88]. Protoplasts are not a popular explant for stable transformation because they are challenging to generate and even more challenging to regenerate [132], but they are attractive for the development of high-throughput genome-editing approaches because electroporation can be automated [133]. Electroporation of microspores and some types of intact plant cells is also possible [134,135]. Wheat microspores have been transformed by electroporation with a plasmid carrying *Cas9* and gRNA genes, but both the transfection efficiency and the genome editing efficiency were low [135].

#### 4.1.5. Other Delivery Methods

Plant cells or embryos can be mixed in a DNA solution containing a suspension of needle-like silicon carbide whiskers, which create holes in the plant cell that allow the uptake of DNA [136]. This method has been used for the transformation of maize [137] but it is not an efficient method [133] and it has not, to our knowledge, been used for genome editing. If target cells or protoplasts can be immobilized, DNA can be delivered to individual cells via thin glass capillaries under a microscope without cell damage [138]. Microinjection requires expensive equipment and is very time-consuming because each cell must be transformed manually [139]. Genome-editing components could theoretically be delivered by microinjection, but regeneration from single transformed cells is not possible in many plant species [139].

### 4.2. RNA

The delivery of genome-editing tools into plant cells as RNA is an interesting approach that has the advantage that RNA has a limited lifespan but can still produce many copies of an encoded protein, such as a sequence-specific nuclease. In the CRISPR/Cas9 system, the gRNA can also be introduced directly as RNA. In ODM, it is necessary to introduce ssODNs or heavily modified DNA/RNA hybrids into the cell, and the principles are similar to those guiding the delivery of RNA. Their delivery is more akin to RNA than DNA and, therefore, is included here.

#### 4.2.1. Particle Bombardment

One of the main drawbacks of particle bombardment for DNA delivery is the frequent low quality of the integration events. This is overcome when using RNA, because no integration event takes place. RNA is more fragile and susceptible to degradation than DNA, particularly plasmid DNA. Nevertheless, it can successfully be delivered into plant cells by particle bombardment.

Zhang et al. [140], for example, bombarded immature wheat embryos with plasmid DNA or in vitro transcribed mRNA representing the CRISPR/Cas9 components and regenerated wheat plants without selection. As anticipated given the higher molecular stability of DNA and the amplification effect of transcription, the DNA-based approach resulted in a higher percentage of mutated plants (3.3%) than the delivery of RNA (1.1%), but a higher percentage of the mutants were homozygous following RNA delivery (35%) compared to DNA (27%). Two of seven homozygous mutants generated by bombardment with DNA were transgene free, compared to all six homozygous mutants generated with RNA (reflecting the inability of RNA to integrate) [140]. Particle bombardment has also been used for ODM in tobacco [141], maize [142], rice [143], and wheat [144] in order to generate herbicide-resistant plants by targeting the *ALS* gene [59].

#### 4.2.2. Protoplast Transfection

Protoplast transfection can also be used to deliver RNA. Stoddard et al. [145] compared the delivery of a TALEN pair targeting the *N. benthamiana ALS* gene as either a DNA construct or in vitro transcribed mRNA. The delivery of DNA achieved a mutation frequency of >70%, whereas mRNA delivery was significantly less efficient (~6%). Because the stability of different mRNA molecules can vary, several combinations of 3′ and 5′ untranslated regions were tested, which doubled the mutation frequency achieved using mRNA. Interestingly, the authors also observed different mutation signatures for DNA and mRNA delivery, even though the same TALEN pair was used: DNA delivery yielded three times as many insertions as mRNA delivery and the insertions were generally >10 bp when the DNA construct was used but <10 bp for mRNA delivery [145]. PEG-mediated protoplast transfection with modified oligonucleotides was also used to generate the Cibus sulfonylurea-tolerant canola line discussed above [146] and confirms that this method is also suitable for ODM.

### 4.3. Proteins and RNPs

The delivery of genome-editing tools, particularly sequence-specific nucleases, in the form of proteins or RNPs is becoming increasingly popular. The high efficiency of these tools allows the targeted changes in the plant genome to be induced within the short half-life of the proteins, and mutated plants can be regenerated without selection. Particle bombardment and PEG-mediated protoplast transfection are the two most commonly reported delivery method for proteins and RNPs, but electroporation, silicon carbide whiskers, and microinjection could also be used in theory.

#### 4.3.1. Particle Bombardment

Svitashev et al. [77] were the first to demonstrate that bombardment can also be used to deliver Cas9/gRNA RNPs to plant cells. They bombarded maize embryo cells with RNPs targeting four different genes and regenerated a surprisingly high proportion of mutated plants without selection (2.4%–9.7%), including 0.3%–0.9% with biallelic mutations. They also compared the on-target and off-target mutation frequencies of the same gRNA delivered as a DNA construct or as a RNP, and found that a comparable proportion of plants was mutated on target (4.0% and 3.7%, respectively) whereas 2% of the plants bombarded with DNA, but not a single plant bombarded with RNPs, had off-target mutations [77]. These findings support earlier studies reporting substantially lower off-target mutation frequencies for RNPs compared to DNA [77]. Efficient genome editing following the bombardment of immature wheat embryos with Cas9/gRNA RNPs has also been reported, with the recovery of 4–5 independent mutants per 100 embryos without selection [126].

#### 4.3.2. Protoplast Transfection

The PEG-mediated transfection of lettuce (*Lactuca sativa*) protoplasts has been achieved with pre-assembled Cas9/gRNA RNPs, and intact lettuce plants were regenerated from edited callus clones [147]. Interestingly, ~6% (2/35) of the callus clones featured monoallelic mutations and 40% (14/35) featured biallelic mutations that were passed on to the T1 generation [147]. The DNA-free delivery of genome-editing reagents is particularly useful for plant species such as grapevine (*Vitis vinifera*) and apple (*Malus domestica*) that do not allow the outbreeding of transgenes. Accordingly, the powdery mildew susceptibility locus *MLO-7* in grapevine and three fire blight susceptibility loci in apple were targeted by transfecting protoplasts with Cas9/gRNA RNPs, and the deep sequencing of genomic DNA extracted after transfection revealed that up to 0.1% of the reads in grapevine and up to 6.7% of the reads in apple contained indels [148]. In petunia (*Petunia* × *hybrida*), the transfection of protoplasts with RNPs achieved mutation frequencies of 5.3%–17.8% in four target genes [149].

## 5. Selection

The delivery of genome-editing reagents to plant cells or tissues is usually followed by a tiered process of selection and/or screening to identify the small proportion of transformants [150,151], the genome-edited events, and among the latter, the events in which the mutations are on-target and have the desired effect. In contrast to conventional plant transformation, genome-editing experiments have two separate outcomes: The presence of the construct expressing the genome-editing tools and the DNA modification itself. Either or both outcomes can be desirable and the applicable screening/selection strategy differs in each case. An efficient selection strategy is particularly important when homologous recombination events must be identified and a double selection strategy may be required to avoid random integration of the donor sequence elsewhere in the genome.

The most appropriate selection strategy depends on the plant species and delivery method. Selectable marker genes that confer antibiotic or herbicide resistance are often used, as well as visual markers and selectable phenotypes. However, most of these strategies require the transfer of a heterologous marker gene into the plant, either positioned alongside the genome-editing components on the same vector, or delivered on a separate plasmid. Although such markers can, in many cases, be removed by outcrossing, the use of marker genes may be undesirable if the new crop variety is intended for commercial exploitation due to the diverse country-dependent definitions and regulations covering genetically modified organisms (GMOs). As an alternative, several DNA-free screening and selection strategies are now available that do not rely on the introduction of marker genes, and these are summarized in Table 1.

### 5.1. Selectable Marker Genes

Marker genes that allow positive selection (selection for the *presence* of the gene) are an important tool in plant breeding because they allow the propagation of transformed plant cells while repressing the growth of the (usually much more abundant) untransformed cells [152]. This is particularly valuable for species and genotypes in which transformation is inefficient [153]. Marker genes that allow negative selection (selection for the *absence* of the gene) are used less often, but are ideal for the identification of homologous recombination events and marker-free plants [154]. Some marker genes allow both positive and negative selection depending on the selection agent. For example, the *dao1* gene encodes a D-amino acid oxidase (DAAO), which can convert certain toxic amino acids into nontoxic ones, allowing positive selection, and can also convert certain nontoxic amino acids into toxic ones, allowing negative selection [155].

In genome-editing applications, positive selectable markers are often introduced along with the other components to allow the selection of cells that are stably transformed with the genome-editing construct. Antibiotic resistance genes are used for this purpose and these are often sourced from bacteria, such as the *E. coli* genes *nptII* (neomycin phosphotransferase, conferring resistance to neomycin and kanamycin) and *hpt* (hygromycin phosphotransferase, conferring resistance to hygromycin) [151]. Whereas antibiotic resistance is usually only needed for the initial selection of transformants and then becomes superfluous, herbicide resistance markers serve a dual purpose because they allow initial selection in the laboratory but also subsequent selective propagation in the field, and on that basis, they may even provide a desirable trait in the final crop [156]. Genetically modified crops often carry markers that confer resistance to phosphinothricin (*bar*, *pat*), glyphosate (*EPSP synthase*), or sulfonylurea herbicides (*acetolactate synthase*) [150,151]. Although antibiotics, herbicides, and other toxic substances are well established for the selection of transformed plant cells, it should be noted that they impose abiotic stress on the cells and can hamper their growth [157].

The preferred selection marker depends on the plant species and genotype. In potato, the *nptII* gene is used almost exclusively for selection because kanamycin resistance allows the recovery of transformed potato plants more quickly than either hygromycin or phosphinothricin [158]. In contrast, kanamycin resistance is rarely used for selection in rice because regeneration is inefficient and the callus tissue appears to have a degree of natural resistance, whereas the *hpt* gene is favored because hygromycin selection distinguishes between untransformed and transformed rice cells more effectively [159,160]. Hygromycin is also commonly used in barley (*Hordeum vulgare*), because it prevents the regeneration of untransformed plants effectively [161,162]. For wheat, a species notoriously recalcitrant to transformation, selection works well with either *bar* or *hpt* [163,164]. Several antibiotic and herbicide resistance genes have been used for the selection of transformed maize plants, but *pat* and *bar*—conveying resistance to phosphinothricin herbicides—are used most often for the development of commercial maize varieties [132,157]. Due to concerns regarding the use of microbial antibiotic or herbicide resistance genes, alternative selection strategies are being developed based on plant-derived genes or non-antibiotic/herbicide selection systems. An example of the latter is the phosphomannose isomerase/mannose selection system, which exploits the fact that mannose is not toxic, but is naturally phosphorylated by plants and inhibits glycolysis. The enzyme phosphomannose isomerase catalyzes the interconversion of mannose-6-phosphate to fructose-6-phosphate and, thus, makes mannose available to the metabolism of transformed cells, allowing the efficient use of this system for selection [150,165].

### 5.2. Visual/Screenable Phenotype

Reporter genes that confer a visible or screenable phenotype are usually non-selectable (i.e., they do not promote or compromise the health of the plant alone or in the presence of a selection agent), but their activity allows transformed and untransformed plants to be identified visually. The visual output can be based on an enzymatic reaction that requires an appropriate substrate (e.g., β-galactosidase, β-glucuronidase, or luciferase [166,167]), the inherent fluorescence of a protein such as green fluorescent protein (GFP) and its derivatives, or a screenable phenotype caused by an introduced transgene (e.g., anthocyanin production [168]), or by changes mediated by the genome-editing tool itself (e.g., a drooping leaf phenotype in rice [169] or a seedless fruit phenotype in tomato [170]). A popular target gene to test the efficiency of sequence-specific nucleases and different delivery methods is phytoene desaturase (*pds*), because a homozygous knockout results in an albino phenotype. The *pds* gene has been targeted in numerous species including rice [171], wheat [172], poplar (*Populus tomentosa* Carr.) [173], cassava [174], and apple [175]. However, because the albino plantlets lack chlorophyll and, thus, cannot survive in soil [176], these mutants are mostly of interest for testing and establishing genome-editing protocols. This issue could be addressed by the simultaneous mutation of the primary target gene and an endogenous gene conferring a screenable phenotype that does not impair plant growth.

### 5.3. High-Throughput Screening of Edited Lines

The emergence of DNA-free methods to edit or engineer plant genomes requires alternative strategies for the identification of edited events without the use of selectable or screenable markers. One approach is high-throughput screening based directly on DNA analysis [140]. The CRISPR/Cas9 components were delivered as DNA or RNA into two hexaploid bread wheat and two tetraploid durum wheat varieties, and plants were regenerated en masse without selection. To identify on-target mutants, a pooled screening approach was used to simultaneously test 3–4 plants regenerated from the same immature embryo in a PCR restriction enzyme assay. Pools that tested positive for mutations were deconvoluted and the plants were tested one by one. Across 11 experiments covering four wheat varieties and seven target genes, the authors identified 1.0%–9.5% mutated wheat plants regenerated from 800–1600 bombarded embryos. Of the T0 mutant plants, 44%–100% were transgene free [140]. It should be noted that many wild-type plants will regenerate alongside the desired mutant plants in the absence of selection. For mutants without a phenotype, this increases the number of plants that must be screened regardless of the efficiency of the employed sequence-specific nuclease. Existing screening methods for marker-free plants regenerated without selection have been developed for marker-free transformation approaches, but cannot necessarily be adapted to screen genome-edited plants generated using DNA-free methods. Marker-free plants are still transgenic and PCR screening can confirm the presence or absence of the transgene, but the small insertions and deletions generated by Cas9 activity are not as easy to identify and some form of sequencing is often required.

## 6. Regeneration

The final, crucial step in any plant transformation protocol is the successful in vitro regeneration of whole plants from transformed plant cells or tissues [177]. For many species and genotypes, the regeneration of transformed plants is a bottleneck because regeneration frequencies are low and the necessary tissue culture steps are time-consuming. There are two main pathways for the regeneration of plants from transformed cells and tissues: Organogenesis (the induction of shoots on the transformed explant and subsequent rhizogenesis, essentially the re-initiation of post-embryonic development) and somatic embryogenesis (the reprogramming of transformed somatic cells to develop into embryos, recapitulating the entire developmental pathway) [177]. Both processes are highly dependent on external stimuli provided by plant hormones (auxins and cytokinins) and exploit the totipotency of individual plant cells, i.e., their ability to change their normal developmental fate and generate all the cells and tissues found in whole plants [178,179]. However, despite the growing body of knowledge concerning the factors that influence regeneration, the interplay is so complex that the optimization of regeneration protocols is still often achieved by trial and error. Insights gained from one species are not necessarily transferrable to other species or even other genotypes of the same species. Protocols for one crop variety can, therefore, only serve as starting point for a related variety [180]. Monocots are usually more challenging to regenerate than dicots, and the undifferentiated monocot meristem cells that can be used for regeneration are thought to be particularly recalcitrant to Agrobacterium-mediated transformation [99]. An interesting alternative approach that sidesteps the need for regeneration was recently described by two research groups using maize haploid inducer lines with CRISPR/Cas9 cassettes for genome editing in elite inbred maize [181,182] and wheat [181] lines. Following haploid induction, the chromosomes of the haploid inducer parent were eliminated and only the edited, elite chromosomes remained. Such techniques are particularly attractive because they enable direct germline editing, avoid tissue culture, and are potentially genotype-independent [181].

### 6.1. Suitable Tissues

The plant tissues used for transformation must not only tolerate the stressful transformation process, but must also contain cells that can dedifferentiate into callus and then undergo organogenesis or somatic embryogenesis to regenerate into whole plants [180]. Embryonic tissues are preferred targets in most crops because their cells have a higher regeneration capacity than the differentiated cells of older tissues [178].

Maize is one of the few monocots that can be transformed rather efficiently by Agrobacterium and particle bombardment (up to 50% efficiency in the presence of a selection marker [133]). Initial maize transformation protocols were based on protoplasts because some maize genotypes can be regenerated from protoplasts into whole plants [183]. However, the lack of efficient and genotype-independent regeneration protocols means that protoplasts are rarely used any more [184]. Most current transformation protocols use immature embryo tissues [132,133]. Svitashev et al. [77] demonstrated a DNA-free genome-editing approach by delivering pre-assembled RNPs into immature maize embryos by particle bombardment, and were able to regenerate mutated plants without selection.

Rice transformation is also quite efficient nowadays, with 50%–90% of callus clones yielding transformants [160] even though rice was initially considered to be a recalcitrant crop [180]. Many rice genotypes can be transformed at least an order of magnitude more efficiently than other cereals [157]. Agrobacterium-mediated transformation is used in most cases [160], but particle bombardment is frequently used in japonica rice cultivars [185]. Immature embryo tissue can be transformed efficiently, but other explants such as scutellum-derived callus [186] have also been used successfully with Agrobacterium. Rice protoplasts are often used to test the efficiency of CRISPR/Cas9 components [50,147,169], but the regeneration of whole plants is still challenging and far from routine [33].

The genetic transformation of wheat was achieved most recently among the major crops [187]. Particle bombardment is the most popular transformation method because it is more efficient and works in more varieties than Agrobacterium, and the latter can trigger cell death in immature wheat embryos [161,188,189]. Regeneration of transformed wheat works best with immature embryo material (the immature inflorescence and the scutellum) [188], but mature embryos are more convenient because they are available throughout the year and the establishment of efficient transformation methods is therefore a key research area [188,190,191]. Particle bombardment has been used to deliver Cas9/gRNA RNPs into immature wheat embryos, and mutated plants were regenerated with a frequency of >4% relative to the number of transformed callus clones [126]. There are no reliable protocols for the regeneration of plants from wheat protoplasts, so in the context of genome editing, protoplasts are mostly used for testing purposes [76]. Microspores are advantageous targets for genome editing because they are haploid and, therefore, carry only half the normal number of target alleles, but neither transformation nor regeneration are very efficient yet [135].

The transformation of barley is usually achieved by mixing immature embryos with Agrobacterium [161]. The barley variety Golden Promise can be transformed with up to 25% efficiency in the presence of a selection marker, but other varieties present more of a challenge [162,192]. The Agrobacterium-mediated transformation of barley ovules is thought to be genotype-independent, but ovule isolation is laborious and requires skilled personnel [192,193]. Efficient genome editing using the CRISPR/Cas9 system has been demonstrated in barley using both Agrobacterium and particle bombardment [194].

Sorghum (*Sorghum bicolor*) is difficult to transform and regenerate [195]. Immature embryos are almost exclusively used as explants for transformation, but despite the optimization of cultivation and transformation conditions, overall efficiencies remain below 20% for particle bombardment and below 10% for Agrobacterium-mediated transformation even in the presence of a selection marker [196]. Using an improved transformation protocol based on an Agrobacterium ternary vector system and the presence of a surfactant, regeneration efficiencies increased to 6%–29% in the popular Tx430 sorghum variety and three African cultivars, allowing the knockout of the centromere-specific histone H3 gene (*Sb-CENH3*) using the CRISPR/Cas9 system [104].

Potato is transformed efficiently by Agrobacterium [110] although transformation and/or regeneration remain a bottleneck in some varieties [197,198]. Popular explants for transformation include leaves from plants cultured in vitro, stem internode segments, and minituber segments [158]. In contrast to most crop species, potato protoplasts can be readily regenerated into plants and have been used for the transient expression of DNA-encoded TALENs [128] or Cas9+gRNA [110] and the DNA-free delivery of Cas9/gRNA RNPs [69], followed in each case by the regeneration of mutated shoots. DNA-free transformation is particularly useful in potato (a tetraploid crop with complex inheritance behavior) because outcrossing the integrated transgene or T-DNA while keeping the desired genetic context can be very difficult [110].

Soybean can be routinely transformed using either Agrobacterium (preferably with cotyledonary node explants) or particle bombardment (preferably with meristems), but the most efficient transformation protocols are not genotype-independent [199,200,201]. A variety of other explant tissues have been tested to identify the best targets for transformation and regeneration [105,202]. Interestingly, two additional low-tech transformation methods have been reported for soybean that deliver DNA directly into the flower, i.e., pollen tube pathway transformation [203] and ovary-drip transformation [204], but it is not clear whether these methods are reproducible [205]. The transformation efficiencies in some soybean genotypes are low and this hampers genome editing and high-throughput genomic analysis [206,207]. ZFNs, TALENs, and the CRISPR/Cas9 system have been used to generate mutations in soybean [67,208,209,210].

Cassava is usually transformed using Agrobacterium with friable embryonic callus as the explant of choice, but despite protocol improvements, it still takes 20–30 weeks to regenerate transgenic shoots and the transformation and regeneration frequencies both remain low [211,212]. Cotyledons are an alternative explant, but the transformation and regeneration frequencies are even lower [212]. Because both genetic transformation and conventional breeding are challenging in cassava, genome editing is of particular interest [213]. Odipio et al. [174] used CRISPR/Cas9 to knock out *pds* in cassava and achieved an unanticipated efficiency of 93%–95% regenerated plants with the associated albino phenotype. In another study, two genes involved in the interaction with a cassava virus were knocked out separately or simultaneously, and even though only 6–15 plants were regenerated per construct, 78% carried biallelic or homozygous mutations in the corresponding target gene [214].

These studies demonstrate that, despite unresolved challenges in the transformation and regeneration of some species, genome editing is a valuable breeding tool because of its high efficiency. This ensures that even small numbers of plants are sufficient to generate the desired mutants.

### 6.2. Timelines

One of the main hurdles preventing the establishment of high-throughput genome-editing protocols for crops is the long overall duration of the transformation and regeneration process, which begins with the cultivation of plants to provide explant material and ends with the harvesting of mature seeds from the transformed T0 generation [195]. The most time-consuming steps are typically the in vitro selection and regeneration of plantlets and the maturation of the regenerated plants. In sorghum, the process takes 9–12 months [195], in maize it takes ~9 months [137], and in wheat—depending on the variety and transformation method—it takes 7–10 months [215,216]. Transformation and regeneration are relatively efficient in rice but the process still takes ~7 months [217,218]. In soybean, it takes 7–9 months when particle bombardment is used for transformation [219]. Efforts to reduce the time required for in vitro cultivation (or to abolish this phase all together) could shorten the overall timelines for the transformation of important crops. Furthermore, increasing the photoperiod of spring wheat, durum wheat, barley, chickpea (*Cicer arietinum*), and pea (*Pisum sativum*) plants to 22 h was recently shown to facilitate the cultivation of six generations in 12 months rather than the usual two or three generations [220].

## 7. Regulatory Aspects of Plant Genome Editing

The regulatory framework that covers genome-edited crops in different countries has a major impact on their development and marketability. In most countries, the current biosafety framework was introduced in the 1980s and 1990s to regulate GMOs generated by conventional gene transfer, and there are ongoing discussions to determine whether the rules are also suitable to regulate products generated by genome editing. Thus far, only Argentina and Brazil have passed supplementary legislation to specifically address the regulatory issues associated with genome-editing applications [221].

There are two major regulatory triggers for GMOs, one based on the process and the other based on the product. The European Union has a process-based regulatory trigger, whereas Canada has a product-oriented evaluation system for plants with novel traits. The USA uses a hybrid system where the regulatory trigger is process-based but the risk assessment is product-based, and genome-edited plants are reviewed on a case-by-case basis [222,223].

**Figure 2:**
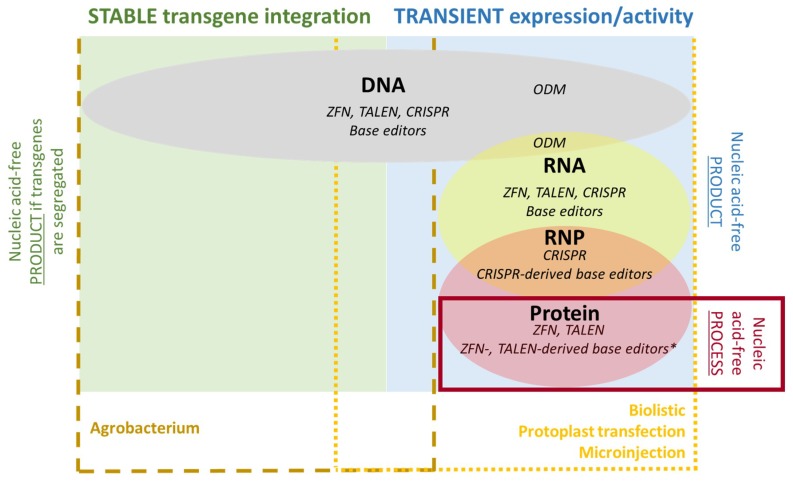
Representation of the relationships between genome-editing tools, delivery methods, and outcomes. The figure shows which tools can be delivered as DNA, RNA, RNPs, or proteins, and which delivery methods are suitable for each cargo type. It also indicates which cargo and delivery methods are available for stable transformation and transient expression, and categorizes them according to the use of nucleic acids (which is relevant for the regulatory assessment of generated plants). The sizes of the shapes are intended to promote visual clarity and do not indicate any relative importance among the methods.

The same genome-edited plant can, therefore, be classified differently from country to country depending on the regulatory framework, making it either subject to or exempt from the rigorous assessment and authorization procedures for GMOs. These aspects must be taken into account when deciding which genome-editing technology to use and how to deliver it (Figure 2). In Argentina, for example, applications for plants that do not retain the transgene in the final product (“null segregants”) are not regulated as GMOs [221]. There are several examples of genome-edited crops in which Agrobacterium-delivered transgenes have been removed by segregation in subsequent generations resulting in transgene-free lines, including the segregation of TALEN-encoding genes and a selection marker from rice with a fragrant phenotype [63], and the segregation of CRISPR components from virus-resistant cucumber plants [71] and powdery mildew-resistant tomato [74].

In process-triggered systems, administrative, legislative, or court decisions are necessary to clarify which genetic modification applications fall under the legislation/GMO definition [221]. On 25 July 2018, the European Court of Justice ruled that organisms developed using genome-editing technology fall under the obligations of Directive 2001/18/EC [224]. This means that genome-edited crops must go through a lengthy and costly comprehensive assessment of indirect and long-term effects, and that they (and food and feed derived from them) must fulfill the requirements for detection, identification, and quantification [221]. In plants with single-nucleotide changes or short indels, it is not possible to determine whether the mutations occurred naturally, were randomly introduced by classical mutagenesis, or were deliberately introduced by genome editing. The market surveillance of seeds or food and feed products will be a serious challenge for international trade between countries with different GMO regulations [225].

## 8. Conclusions and Outlook

Genome editing in plants became possible when the first designer nucleases were described in the late 1990s, but the field has exploded in popularity since 2012 following the development of the CRISPR/Cas9 system. Depending on the desired outcome, editing can be achieved using sequence-specific nucleases, ODM, or base editors. The easiest approach, and therefore the most widely reported, is the use of sequence-specific nucleases to disrupt genes and generate knockout mutations. However, the range of qualitative traits of agricultural interest that can be engineered by gene disruption is rather limited. For many traits, especially quantitative traits, the modulation of gene function or expression is more desirable. This has been achieved by converting sequence-specific nucleases into transcriptional regulators [226], and by the direct editing of cis-acting regulatory sites within genes, as recently demonstrated in tomato [227]. Although this approach requires more development and screening efforts to identify and evaluate the desired phenotypes, the modification of non-coding sequences and the engineering of quantitative trait variation are likely to be the next steps in genome-editing applications for commercial crops.

In addition to the mutation of DNA sequences, the properties of plants could also be modified by epigenome editing, which involves the control of gene expression by targeting DNA methylation or histone acetylation [228,229]. It could have important applications in agriculture given that no genetic modification is involved and it would not be subjected to GMO regulations, at least in product-based regulatory frameworks. Epigenome editing could be used not only to modulate the expression of specific genes or gene families, but also to influence the chromatin landscape of selected genomic regions [230]. However, before epigenome editing can be used to produce new crop varieties, more research is needed to evaluate the stability and heritability of the epigenetic changes introduced by new epigenome-editing tools.

One of the major technical hurdles associated with genome editing in plants is the dependence on tissue culture, which dictates the explants that can be used for the delivery of genome-editing constructs or components, affects the efficiency of that delivery process, and determines whether whole plants can be regenerated. To exploit the tremendous potential of genome-editing technologies, it is necessary to study gene function on a genomic scale allowing the identification of candidate genes underlying agronomic traits. In addition, to ensure a high editing efficiency, several enzymes or guide RNAs should be tested for each gene. To achieve this, much greater effort is required to develop efficient protocols for the transformation and regeneration of diverse species and varieties, preferably in an automated high-throughput manner. Direct modification of the germline or meristematic tissues in planta would bypass the tissue culture steps and overcome the species- and variety-dependent limitations of the regeneration process. This would enormously simplify and accelerate the creation of genome-edited lines, and the first progress in this direction has already been made. Very recently, researchers at Syngenta achieved genome editing in maize by combining the delivery of the *Cas9* transgene by pollen with haploid induction [181].

Although genome-editing technology is already mature enough to allow the generation of edited lines for commercial use, there remains a need to continuously improve and optimize all genome-editing approaches. Now that genome editing is finally a reality, it is clear that sociopolitical rather than technological factors will restrict the widespread adoption of genome-edited crops. When genetic modification involves the introduction of heterologous DNA to confer a new function, it is clear that both the process and the product fall under GMO regulations. However, the modification of one or a few bases can be achieved by a spectrum of processes, including natural mutation, random chemical/radiation mutagenesis, and deliberate editing with or without the introduction of DNA. Given that nucleic acid-free genome-editing methods are the least likely to trigger regulatory and safety concerns, they will continue to receive significant attention. In particular, the efficient delivery of proteins and RNPs should be optimized, and effective nucleic acid-free ways to identify cells containing these reagents should be established.

Although current genome-editing technologies are far more precise than traditional random mutagenesis, the acceptance of genome-edited crops remains a major bottleneck. A limited understanding of the underlying technical aspects and outcomes, together with the common misbelief that exemption from GMO regulations, would mean the uncontrolled release of new edited crops, contributing to public suspicion and opposition in many countries, particularly in Europe. This, in turn, influences the political decisions of the authorities, even those outside Europe. Once all the technical hurdles have been tackled and a genome-edited crop is finally ready to go on the market, it must comply with the legal obligations including labeling and traceability, which differ from country to country. As it is impossible to determine the process by which point mutations have been generated, international trade and market surveillance for genome-edited products will be a significant challenge [225]. We will be soon facing these issues in practice, so the harmonization of regulations on a global level is urgently required.

## Figures and Tables

**Table 1:** Examples of selection-free genome editing in different plant species.

Delivery Method	Cargo	Plant Species	Tissue	Selection	Mutation Efficiency	Calculation	Reference
Agrobacterium-mediated transformation	DNA (transient) CRISPR/Cas9	Tobacco *Nicotiana tabacum*	Leaf disks	No	2.57%	Mutated plants/total regenerated shoots	[107]
				No	17.2%	Non-transgenic plants/total mutant plants	
Particle bombardment	DNA CRISPR/Cas9	Wheat *Triticum aestivum*	Immature embryos	No	3.3%, 2/26 plants homozygous and transgene-free	Mutated plants/bombarded embryos	[140]
	IVT mRNA CRISPR/Cas9			No	1.1%, 6/17 plants homozygous and transgene-free	Mutated plants/bombarded embryos	
Particle bombardment	RNP CRISPR/Cas9	Maize *Zea mays*	Immature embryos	No	2.4–9.7%, 9.6–12.9% of mutated plants biallelic	Mutated plants/analyzed plants	[77]
Particle bombardment	RNP CRISPR/Cas9	Wheat *Triticum aestivum*	Immature embryos	No	4.4%	Mutated plants/bombarded embryos	[126]
Protoplast transfection (PEG)	DNA TALEN	Potato *Solanum tuberosum*	Protoplasts	No	11–13%	Mutated callus/total protoplast-derived callus	[128]
Protoplast transfection (PEG)	DNA TALEN	Tobacco *Nicotiana benthamiana*	Protoplasts	n.a.	70.5%	Deep sequencing of protoplasts	[145]
	mRNA TALEN			n.a.	5.8–16.9%	Without/with UTR	
Protoplast transfection (PEG)	RNP CRISPR/Cas9	Lettuce *Lactuca sativa*	Protoplasts	No	46%, 6% mono-, 40% biallelic	Mutated callus/analyzed callus	[147]
Protoplast transfection (PEG)	RNP CRISPR/Cas9	Grapevine *Vitis vinifera* cv. Chardonnay	Protoplasts	n.a.	0.1%	Deep sequencing of protoplasts	[148]
		Apple *Malus domestica* cv. Golden delicious	Protoplasts	n.a.	0.5–6.7%	Deep sequencing of protoplasts	
Protoplast transfection (PEG)	RNP CRISPR/Cas9	Petunia *Petunia x hybrida*	Protoplasts	n.a.	5.3–17.8%	Deep sequencing of protoplasts	[149]
